# False memory and COVID-19: How people fall for fake news about COVID-19 in digital contexts

**DOI:** 10.3389/fpsyg.2022.972004

**Published:** 2022-10-13

**Authors:** Ivan Mangiulli, Fabiana Battista, Nadja Abdel Kafi, Eline Coveliers, Theodore Carlson Webster, Antonietta Curci, Henry Otgaar

**Affiliations:** ^1^Department of Education, Psychology, Communication, University of Bari Aldo Moro, Bari, Italy; ^2^Faculty of Law and Criminology, Leuven Institute of Criminology, Leuven, Belgium; ^3^Forensic Psychology Section, Faculty of Psychology and Neuroscience, Maastricht University, Maastricht, Netherlands; ^4^School of Applied Sciences, Psychology, Edinburgh Napier University, Edinburgh, United Kingdom

**Keywords:** false memories, COVID-19, fake news, conspiratorial content, individual differences

## Abstract

People are often exposed to fake news. Such an exposure to misleading information might lead to false memory creation. We examined whether people can form false memories for COVID-19-related fake news. Furthermore, we investigated which individual factors might predict false memory formation for fake news. In two experiments, we provided participants with two pieces of COVID-19-related fake news along with a non-probative photograph. In Experiment 1, 41% (*n* = 66/161) of our sample reported at least one false memory for COVID-19-related fake news. In Experiment 2, even a higher percentage emerged (54.9%; *n* = 185/337). Moreover, in Experiment 2, participants with conspiracy beliefs were more likely to report false memories for fake news than those without such beliefs, irrespective of the conspiratorial nature of the materials. Finally, while well-being was found to be positively associated with both true and false memories (Experiment 1), only analytical thinking was negatively linked to the vulnerability to form false memories for COVID-19-related fake news (Experiment 2). Overall, our data demonstrated that false memories can occur following exposure to fake news about COVID-19, and that governmental and social media interventions are needed to increase individuals’ discriminability between true and false COVID-19-related news.

## Introduction

The COVID-19 pandemic has been adversely affecting people’s lives in several ways since its outbreak in 2020. This pandemic is now noted as a traumatic event, leading people to experience negative and unpleasant emotions that could exert a downside influence on their mental health (Canet-Juric et al., 2020; Sanchez-Gomez et al., 2021). Despite its massive consequences on people’s well-being (Vindegaard and Benros, 2020), the COVID-19 pandemic also represents a major threat for how people remember, share, and report information surrounding the disease in itself.

The World Health Organization (WHO) declared the COVID-19 pandemic as an “infodemic” (WHO, 2020), implying that people are exposed to a copious quantity of misleading information in the form of fake news, particularly amplified by social media, which overlaps or interferes with official communications. Fake news is described as “fabricated information that mimics news media content in form but not in organizational process or intent” (Lazer et al., 2018, p. 1094). During the pandemic, 79% of UK and 72% of USA citizens used the internet (including social media) to look for COVID-19-related information (Nielsen et al., 2021). Yet some of that information was likely intermixed with fabricated news (Greenspan and Loftus, 2021). This, in turn, could have made people struggle to differentiate between true and fabricated COVID-19-related news, rendering them vulnerable to falsely remembering having heard/seen these fake news stories (Greene and Murphy, 2020). Furthermore, several studies have shown that individual’s factors may underlie this vulnerability to form false memories. For instance, lower cognitive ability and lower analytical thinking relate to a stronger false memory formation for fabricated events (Zhu et al., 2010; Murphy et al., 2019; Greene et al., 2021).

Accordingly, the main goal of the present experiments was to investigate whether people fall prey to false memory formation when exposed to COVID-19-related fake news. In addition, to better understand why people may report false memories for COVID-19-related information, we examined whether individual factors (e.g., well-being, cognitive abilities, and analytical thinking) may predict false memory formation.

### Fake news and false memory formation

Different methods exist to study the likelihood to report both spontaneous and suggestive false memories. False memories for fake news can be categorised as suggestive false memories due to the external “pressure” of the fake information. One of the most influential paradigms used to study suggestive false memories is the misinformation paradigm (Loftus, 2005). A wide variety of research using this paradigm has shown that when people are exposed to misleading post-event information, they tend to report this information into later memory tests (i.e., misinformation effect; see Loftus, 2005). Simply put, when people are presented with an event, and then are given false information about it, they frequently report false post-event information in their memory accounts for that episode (Wylie et al., 2014; Nichols and Loftus, 2019).

From a theoretical perspective, false memories are explained by relying on principles drawn from the source monitoring framework (SMF; Johnson et al., 1993). According to the SMF, people make certain attributions when retrieving an experience. Specifically, when a mental representation contains many phenomenological characteristics (e.g., perceptual, auditory), people tend to attribute it to an experienced event thereby confusing the source of this representation. Such source monitoring errors can also arise when the retrieval of misinformation contains memory qualities as the experienced event (e.g., perceptual, emotional, or contextual) which could lead to incorrectly allocating it to the original source, resulting in the fake information being reported as part of the original experience (Johnson et al., 1993; Mitchell and Johnson, 2000).

Recently, interest has shifted towards the examination of how fake news can affect the production of false memories. For instance, Murphy et al. (2019) examined false memories formation for fabricated events during the Ireland’s abortion referendum in 2018. Participants received two fake and four true news accompanied by pictures showing some of the campaign activities. Almost half of the participants (48%) eventually reported a false memory for at least one of the fake events. Moreover, results from this study showed that faked news accounts were in line with people’s already existing political standpoints. Indeed, participants in favour of legalizing abortion were more likely, than those against it, to remember fabricated news about the campaign against abortion and vice versa (see also Greene et al., 2021).

Of interest for the current work, Greene and Murphy (2020) investigated individual differences (i.e., objective COVID-19 knowledge, analytical thinking) in people’s vulnerability to form false memory for COVID-19 related fake news. Participants were exposed to six COVID-19 news stories (four true and two false) accompanied by a non-probative photograph. Then, participants were asked whether they remembered having heard/seen all the news. Almost a quarter of participants (22.56%) reported a false memory for at least one fake story. Also, the authors found that certain individual’s factors were associated with false memory formation. For instance, while objective COVID-19 knowledge was positively related with fewer false memories, higher levels of analytical thinking were related with fewer memories for both real and fake news stories.

Additionally, Scuotto et al. (2021) examined whether individuals variables (e.g., COVID-19 perceived and objective knowledge, fear of the disease) affected people’s COVID-19-related false memory creation. The authors showed an Italian sample of university students eight COVID-19-related news stories each accompanied by a picture. Four out of the eight news stories were carefully fabricated. In line with the work conducted by Greene and Murphy (2020), Scuotto et al. (2021) found that participants correctly recalled more true than fake news stories, although about 19% of the entire sample remembered having seen/heard a COVID-19 fake event that has never took place. Furthermore, higher levels of objective knowledge, as well as a greater fear that loved ones would contract COVID-19, were associated with a decrease in false memories. Taken together, these results suggest that susceptibility to form false memories for fabricated events could be influenced by several individual’s differences.

### Individual factors, fake news, and false memories

Several studies have examined individual’s difference that might affect how fake news leads to false memory production. For instance, Murphy et al. (2019) showed that low cognitive ability increased the likelihood that fake news is remembered as true (see also Greene et al., 2021). Moreover, in addition to Greene and Murphy (2020), research demonstrated that obtaining high scores on analytic thinking goes hand in hand with an ability to distinguish between true and false headlines (Pennycook and Rand, 2019), even about COVID-19 (Pennycook et al., 2020; but see Scuotto et al., 2021). Overall, these results are in line with work pointing out that analytical thinking can guard against the acceptance of fake news and hence might mitigate against COVID-19 misinformation (Pennycook and Rand, 2019; Pennycook et al., 2020).

Although analytic thinking might help to immunize against false memories for fake news, existing perceived knowledge and self-interest about a certain topic might catalyse false memories for fake news stories (Castel et al., 2007; Mehta et al., 2011; O’Connell and Greene, 2017). This is more likely to occur when people overestimate their knowledge (a phenomenon called “overclaiming”; see Atir et al., 2015) or strongly engage with a certain topic. Perceived knowledge and self-interest may make participants reluctant to admit ignorance about topic-related events, increasing the tendency to report a memory for a given story irrespective of it being fabricated or not. Considering that people look for news about COVID-19 to varying degrees (Nielsen et al., 2021), these differences in engagement might also predict people’s susceptibility to false memory. Yet in contrast to what previously revealed (e.g., O’Connell and Greene, 2017), Greene and Murphy (2020), and Scuotto et al. (2021), recently showed that more knowledge about COVID-19 caused a reduction in false memory creation.

However, not all the knowledge circulating about specific topics is in line with scientific evidence, and yet people show some interest for it. The COVID-19 pandemic stimulated the spread of a plethora of fake information that was sometimes characterized by conspiratorial contents (Quinn et al., 2020; Stein et al., 2021). Claims such as that SARS-CoV-2 virus is being used as a biological weapon, or that 5G^1^ has caused the virus, became popular claims within the general population. Research suggested that conspiracy theories are more likely to be accepted during stressful time, such as crisis situations (Van Prooijen and Douglas, 2017; Oleksy et al., 2020). The explanation here is that such situations are typically marked by feelings of uncertainty, lack of control, and fear. When these feelings come to play, conspiracy theories usually provide people with simple but bizarre explanations, leading them to attribute malevolent meaning to events that are unexpected and chaotic (e.g., a pandemic). In addition, research showed that people are not opposed to believing in theories that are entirely fabricated and to endorsing fake conspiratorial materials (Swami et al., 2011; Anthony and Moulding, 2019). Also, people are more vulnerable when fake information aligns with their beliefs (Frenda et al., 2013). Even though it is not known yet, one might argue that people who believe in conspiracy theories could be even more susceptible to report a false memory for fake news that enclose conspiratorial contents.

### The current experiments

In two experiments, we examined whether people could form false memories for COVID-19 fake news. We showed participants six COVID-19-related events (four true and two false) accompanied by non-probative photographs. In addition, in Experiment 2 we provided participants with fake news containing either conspiratorial content or not. In both studies, we asked them whether they remembered having heard/seen those events. For both experiments, in line with previous work on fake news and false memory (Murphy et al., 2019; Greene and Murphy, 2020; Greene et al., 2021; Scuotto et al., 2021), we expected that a non-trivial percentage of participants (≈ 19–48%) would report a false memory for at least one COVID-19-related event that has never occurred. Furthermore, for Experiment 2, we predicted that participants believing in conspiracy theories would report more false memories with conspiratorial content than those who did not believe in conspiracy theories.

A subsidiary aim was to elucidate which factors predict susceptibility to false memory for COVID-19-related fake news. To examine this, in both experiments we examined several factors that could potentially interact with the link between fake news and false memories. In Experiment 1, we assessed the effects of individuals’ well-being and health risk perception connected to COVID-19 on the vulnerability to form false memory. The rationale to test these factors is because the COVID-19 pandemic has caused a long-lasting period of emotional distress that affects people’s life, in terms of anxiety, infection fears, frustration, stigma, and financial loss (Brooks et al., 2020). Such states, along with people’s COVID-19 health risk perception (see Lanciano et al., 2020), could play a role in remembering COVID-19-related events. However, there is currently no research that investigated individuals’ well-being and health risk perception connected to false memories for COVID-19-related materials. Yet some hints originate from previous work (Greene and Murphy, 2020), wherein they found that anxiety levels were positively associated with reporting true, but not false memory. Indeed, Scuotto et al. (2021) did not show an higher false memory rate in individuals suffering from anxiety. Of course well-being, health risk perception, and anxiety are distinct factors, which could be linked to each other (e.g., those who perceived high risk of being infected by COVID-19 could feel more anxious, perhaps affecting their overall current well-being). Hence, we expected that both higher individual’s well-being and health risk perception would be linked to higher levels of true but not false memory levels.

In Experiment 2, in accordance with prior studies showing that false memories creation is related to individual cognitive differences (e.g., Zhu et al., 2010; Battista et al., 2020; Scuotto et al., 2021), we hypothesised that a higher level of cognitive abilities and analytical thinking would be related to high rates of true memory, but not lower amount of false memory. Finally, we predicted that existing knowledge, self-interest, and conspiracy beliefs would be associated with an increased tendency to report both true and false memory.

## Experiment 1

### Method

#### Participants and design

We performed an *a priori* power analysis using G*Power (i.e., *t*-tests, difference between two dependent means, one-tail; Faul et al., 2007) with a power of 0.80 and an effect size of *dz* = 0.25 (*α* = 0.05). This analysis indicated that a total of 101 participants was needed. We recruited 186 Flemish participants using a snowball sampling technique (Goodman, 1961). We eliminated data from 25 participants because they did not either fully complete the experiment or failed one of the attention checks. Hence, we performed analyses on a total of 161 participants (range: 18–77, *M* = 35.39, *SD* = 18.34; 64.6% female). We employed a within-subjects design, with true and false memory rates as main dependent variables. This experiment was approved by the ethical committee of KU Leuven (G-2020-2,781). The data set and materials can be found on the Open Science Framework (OSF; osf.io/3xhkt).

### Materials and measures

#### True and fake news

A total of 6 COVID-19-related news stories were presented to participants. Irrespective of being true or false, the news was accompanied by a non-probative photograph. Each participant was asked to choose one of the following options once exposed to the news: “*I have a clear memory for seeing/hearing about this*,” “*I have a vague memory for this event occurring*,” “*I do not have a memory for this, but it feels familiar*,” “*I remember this differently*,” “*I do not remember this*.” We then dichotomized their responses in either having memory for the event (i.e., having heard/seen the news) or not in line with previous work (e.g., Greene and Murphy, 2020). That is, participants’ answers to both true and fake news “*I have a clear memory for seeing/hearing about this*” or “*I have a vague memory for this event occurring*” were categorized as for “Having memory for the event,” while the rest of answers (i.e., “*I remember this differently*,” or “*I do not remember this*”) were categorized as for “Not having memory for the event.” Thus, the response “*I do not have a memory for this, but it feels familiar*” was not categorised to account for individual differences in familiarity.

Four news stories referred to true events, whereas the remaining two involved false events. The true news stories depicted real COVID-19 situations that occurred during 2020–2021. They concerned (i) a famous Belgian virologist lashing out on Twitter^2^, (ii) an 8-year-old Belgium child who underwent intensive care, (iii) Donald Trump and (iv) Brazil’s President, Jair Bolsonaro, underestimating the effects of COVID-19. All the true news are fully reported in Supplemental Materials (SM1). The two COVID-19-related fake news stories were the following:

1. Three Belgian coffin carriers shared the photo on Facebook of a storage area, filled with coffins. The news was about 300 people who died from COVID-19 and still had to be buried. The three Belgians distributed this photo with the intention to make people aware of COVID-19 consequences: “Because of my job, I get in touch with the consequences of COVID-19 every day. Only then you understand how serious the situation is,” wrote one of them.
2. A large online survey from the University of Antwerp showed that 60% of the students considered COVID-19 to be “just a cold” or a “minor flu.” A number of the students also indicated that they found the restrictive measures excessive. There were also a small number of students who did not believe that COVID-19 really exists. Virologists were shocked when reading the results of the university survey.

#### World Health Organisation: Five well-being index (WHO-5)

The WHO-5 is a self-report measure to assess current wellbeing (WHO, 1998). Translated in more than 30 languages, the WHO-5 has been found to adequately screen for mental issues (e.g., depression) as well as for outcome in clinical trials. It has a good construct validity as a unidimensional scale measuring well-being (see Topp et al., 2015). Participants are requested to rate 5 statements (e.g., “*I have felt cheerful and in good spirits*”) on a 6-point-Likert scale, ranging their answers from 0 (“At no time”) to 5 (“All of the time”). The total raw score (range: 0–25) is multiplied by 4 to give the final total score, with 0 representing the worst imaginable well-being and 100 the best (*α* = 0.88, *95%CI* [0.84, 0.90]).

#### Health risk perception

Participants were asked to indicate to what extent they think they were in a high risk group for COVID-19 (i.e., with a higher-than-expected risk for developing COVID-19), ranging their answers from 1 (“Definitely I am a person with a high risk”) to 5 (“Definitely I am not a person with a high risk”).

#### Procedure

The entire experiment was conducted in Dutch, and online using Qualtrics.^3^ After signing the informed consent and completing demographic information, participants were exposed to the 6 news (i.e., 4 true and 2 fake), and asked to rate their memory for having heard/seen them. Subsequently, they completed both the WHO-5 and the high risk perception questionnaire. Furthermore, participants were asked two questions concerning their opinion and behaviour about COVID-19^4^ (i.e., “*What’s your point of view about COVID-19?*” and “*To what extent do you adhere to the current COVID-19 measures*”?). The sequence of the 6 news, as well as the subsequent questions and measures adopted, were randomized across participants. Finally, participants were thanked and debriefed.

### Results and discussion

#### True and false memory rate

In line with previous work (Greene and Murphy, 2020), we dichotomized their responses in either having memory for the event (i.e., having heard/seen the news) or not to obtain a nuanced understanding on participants’ true and false memory rates. With respect to true events, participants on average correctly reported to having heard/seen 1.88 (*SD* = 1.14) out of the 4 news presented. Of importance, participants falsely recognized having heard/seen 0.66 (*SD* = 0.69) out of the 2 fake news presented. Moreover, 20.7% (*n* = 29/140) of the participants falsely reported having heard/seen the first fake news story (i.e., 300 coffins), while 40.5% (*n* = 53/131) falsely recognized having heard/seen the second one (i.e., university survey). Thus, overall, about 41% of the participants (*n* = 66/161) reported at least one false memory after being exposed to a fake news. Table 1 displays participants’ response for both true and fake news.

**Table 1:** Participants’ responses to true and fake news (Experiment 1).

Response	True news			
	van Ranst’s tweet	8-years old boy	Donald Trump	Jair Bolsonaro
I have a clear memory for seeing/hearing about this	24.8% (*n* = 40)	16.1% (*n* = 26)	12.4% (*n* = 20)	36% (*n* = 58)
I have a vague memory for this event occurring	26.1% (*n* = 42)	23.6% (*n* = 38)	26.1% (*n* = 42)	23% (*n* = 37)
I do not have a memory for this, but it feels familiar	21.7% (*n* = 35)	16.1% (*n* = 26)	15.5% (*n* = 25)	9.9% (*n* = 16)
I remember this differently	3.1% (*n* = 5)	1.9% (*n* = 3)	2.5% (*n* = 4)	3.1% (*n* = 5)
I do not remember this	24.2% (*n* = 39)	42.2% (*n* = 68)	43.4% (*n* = 70)	28% (*n* = 45)
Average true memory rating [mean (SD)]	0.47 (0.50)	0.46 (0.50)	0.65 (0.47)	0.66 (0.47)
	**Fake news**			
	**Three-hundred coffins**		**University Survey**	
I have a clear memory for seeing/hearing about this	5.6% (*n* = 9)		7.5% (*n* = 12)	
I have a vague memory for this event occurring	12.4% (*n* = 20)		25.5% (*n* = 41)	
I do not have a memory for this, but it feels familiar	13% (*n* = 21)		18.6% (*n* = 30)	
I remember this differently	6.8% (*n* = 11)		8.7% (*n* = 14)	
I do not remember this	62.1% (*n* = 100)		39.8% (*n* = 64)	
Average false memory rating [mean (SD)]	0.21 (0.40)		0.40 (0.49)	

#### Predictors of true and false memory for COVID-19 news

We performed multiple regression analyses for both true and false memory rates, with predictor variables (1) well-being (range: 0–92; *M* = 47.27, *SD* = 21.49), and (2) high risk [78.9% (*n* = 127/161) being not high risk people; *M* = 4.11, *SD* = 1.38]. According to previous work (Greene and Murphy, 2020), true memory data were examined using linear regression and Poisson regression was adopted to analyse false memory rates.^5^ Both models showed that the variables predicted the outcome variables: True memory, *R^2^* = 0.108, *F*(2,158) = 9.55, *p* < 0.001, and false memory counts, *χ^2^*(2) = 7.87, *N* = 161, *p* = 0.02. Specifically, higher well-being levels were associated with an increased inclination to report both true and false memories for COVID-19 news, both yielding positive effect sizes. This means that for every 1-unit increase in well-being levels, both true and false memories increased by 1.018 and 1.015 units, respectively (see Table 2). By contrast individuals’ health risk perception was not statistically linked to reporting both true and false memories, and thus deemed non-relevant, even though effect size for the latter rate was found to be positive [*exp(B)* = 1.222; see Table 2].

**Table 2:** Regression coefficients for predictors of vulnerability to COVID-19 news (Experiment 1).

Predictor	*B*	*SE (B)*	*β^+^*	*t*	*p*	95% CI (*B*)	
						Lower	Upper
True Memory Rate							
(Constant)	1.06	0.375		2.48	0.005	0.327	1.80
Well-being*	0.017	0.004	1.018	4.21	0.000	0.009	0.026
Health risk perception	−0.002	0.064	0.0998	−0.030	0.971	−0.129	0.124
	*B*	*SE (B)*	*β^+^*	*Wald χ^2^*	*p*	95% CI (*B*)	
						Lower	Upper
False Memory Rate							
(Intercept)	−2.52	0.680	0.080	13.78	0.000	−3.86	−1.19
Well-being*	0.016	0.006	1.015	5.94	0.015	0.003	0.028
Health risk perception	0.201	0.109	1.222	3.34	0.068	−0.015	0.416

*p  < 0.05; ^+^For linear regressions (i.e., true memory rate), β is given as 0 for no effect with values <0 for negative effects and >0 for positive effects. Whereas for Poisson regressions (i.e., false memory rate), β [i.e., exp(B)] is given as 1 for no effect, with values >1 for positive effects and <1 for negative effects.

To sum up, a nontrivial percentage of participants (41%) reported having heard/seen at least one COVID-19 fake news event. This is in line with previous research (e.g., Murphy et al., 2019; Greene and Murphy, 2020). For one thing, research showed that the emotional content of an information increases susceptibility to false memory. Studies comparing different emotional materials (positive, negative, and neutral) typically found that false memory rate is highest for negative information (Porter et al., 2003; Otgaar et al., 2008; Van Damme and Smets, 2014; Zhang et al., 2021; see also Bookbinder and Brainerd, 2016). Thus, our findings stress the ease with which memories for fake news can be formed concerning a stressful, emotional episodes, such as pandemic-related events.

Of interest are the results concerning the association between well-being and COVID-19 false memory. In our experiment, a moderate well-being state was linked with a propensity to report both true *and* false memories. It could be the case that extreme relevance of COVID-19 for the individual’s well-being induced a sort of attentional bias so that people recognized all news (both true and false) as true. Thus, because of the long-lasting pandemic period, when well-being states are not optimal and people are subjected to COVID-19 materials, they are likely to remember having heard/seen any COVID-19 news irrespective of being true or false. Unexpectedly, health risk perception to COVID-19 was associated neither with true nor false memory for COVID-19-related materials. Arguably, this lack of association was due to the fact that the risk perception of being infected in our sample was quite low, thereby not affecting the way people remembered COVID-19-related news. Furthermore, and relatedly, Scuotto et al. (2021) showed that it was more the fear of loved ones contracting COVID-19, rather than contracting it personally, that might reduce false memory formation.

## Experiment 2

In Experiment 2, we extended our work on fake news and false memories. Some changes were done in this experiment. First of all, we showed COVID-19 fake news with either conspirational content or not. Furthermore, we focused on additional variables associated with false memory (e.g., cognitive abilities, analytical thinking, knowledge, and self-interest), in order to reveal commonalities, and possible discrepancies, with prior research on COVID-19 and fake news (e.g., Murphy et al., 2019; Greene and Murphy, 2020; Scuotto et al., 2021).

### Method

#### Participants and design

An *a priori* power analysis using G*Power (i.e., t-tests, difference between two independent means, one-tail; Faul et al., 2007), with a power of 0.80 and an anticipated medium effect size of *d* = 0.30 (*α* = 0.05), indicated 278 participants. We recruited a total of 337 people (range: 18–75, *M* = 36.05, *SD* = 12.78; 46.6% female). Participants were recruited *via* Amazon Mechanical Turk (MTurk)^6^ and through university advertisements. Those enrolled *via* MTurk received a financial reimbursement of 1 dollar, whilst university students received a research credit as compensation for participating in the current experiment. No data from any participant was excluded after checking the attentional questions. We used a between-subjects design [conspiratorial content (*n* = 165) vs. no conspiratorial content (*n* = 172)], with true and false memory rates as main dependent variables. This experiment received approval by the ethics review committee of Maastricht University (ERCPN-Marble 229_118_10_2020). Experiment 2 was pre-registered (osf.io/w78vf), and the data set and materials can be found on OSF (osf.io/3xhkt).

### Materials and measures

#### True and fake news

All participants were exposed to a total of 6 COVID-19-related news accompanied by a non-probative photograph (i.e., 4 true and 2 fake news). Participants options to rate their memory for having heard/seen the news were the same as in Experiment 1. Also in Experiment 2, we dichotomized participants’ responses in either having memory for the event (i.e., having heard/seen the news) or not (e.g., Greene and Murphy, 2020). For the true news stories, we provided participants with those that were shared worldwide. Specifically, participants were randomly given 4 out of 8 true events. True news were about (i) new technologies in Taiwan to contrast the pandemic, (ii) a tiger testing positive to the virus, (iii) New York’s hospitals overwhelmed with COVID-19 patients, (iv) Russian’s first vaccine, (v) Italian army helping crematories in Bergamo (Italy), (vi) Donald Trump and the first lady testing positive to COVID-19, (vii) Italian people singing from balconies during lockdown, and (viii) Olympic games in Japan postponed due to the pandemic. All the COVID-19 true news are entirely shown in Supplemental Materials (SM2). Furthermore, we showed participants 2 fake news. Of importance, we created two versions for each fake news, either containing conspiratorial content (1a and 2a) or not (1b and 2b). Each participant received one version per fake news, which were counterbalanced:

1a. Currently, various possible future COVID-19 vaccines are being tested on participants. Thus far, however, almost all of them have evoked serious side-effects in test persons. Yet certain pharmaceutical companies have stated already, that they still aim at making these vaccines available.^7^
1b. Currently, various possible future COVID-19 vaccines are being tested on participants. Thus far, however, almost all of them have evoked serious side effects in test persons.
2a. Many countries have implemented COVID-19 warn apps in order to decrease the number of infections. Computer specialists from Norway could reveal that many European governments use these apps in order to increasingly control their citizens.
2b. Many countries have implemented COVID-19 warn apps in order to decrease the number of infections. Yet many of these apps have been demonstrated to display severe data leakages.

#### Wordsum test

The Wordsum test is a ten-item subtest of the Wechsler Adult Intelligence Scale vocabulary test use to evaluate people’s cognitive ability (Wechsler, 2008). Its high correlation with broad test of general intelligence makes it an acceptable tool for assessing general cognitive ability (Meisenberg, 2015). When presented with a target item (e.g., “Space”), participants are asked to choose the closest match from a list of four other words (e.g., “Room”). For each correct response participants are assigned one point (range: 0–10). Participants’ average score is usually 6 correct responses out of 10 (Meisenberg, 2015; *α* = 0.80, *95%CI* [0.76, 0.83]).

#### Cognitive reflection test

Participants were assessed on a six-item Cognitive Reflection Test (CRT), which is a frequently used tool in research into heuristics and biases, and overall analytical thinking (Frederick, 2005). Three of the included test items were of the general numeric version of the CRT and the other three items pertained to a non-numeric version of the test (Thomson and Oppenheimer, 2016). This combination has already been used in previous studies and shown to correlate significantly (Thomson and Oppenheimer, 2016; Pennycook and Rand, 2019). An example of a possible CRT item was “*The ages of Mark and Adam add up to 28 years total. Mark is 20 years older than Adam. How many years old is Adam?*.” The intuitive answer of 8 is wrong and indicates that no reflective reasoning had been involved in the search for an answer (Pennycook and Ross, 2016; Pennycook and Rand, 2019; *α* = 0.82, *95%CI* [0.78, 0.85]).

#### Knowledge about COVID-19

In line with previous work (e.g., Greene and Murphy, 2020), we developed a 10-item knowledge test concerning COVID-19 to tap into participants’ general knowledge surrounding the virus. Participants were given the possibility to choose the correct option among four possible choices. Items were based on current information at the time of conceiving the test (i.e., January–February 2021). For instance, to the item “*Which country had announced the first lockdown to reduce the spread of the corona virus?*” participants could answer among (a) Italy, (b) China, (c) Spain, and (d) Ireland. Other items included questions on health-protective behaviors, the incubation period, and lockdowns in different countries (*α* = 0.57, *95%CI* [0.50, 0.63]).

#### Interest in COVID-19

For the assessment of level of interest, participants indicated how much they engaged with the topic of COVID-19 on social media, television, newspaper articles, radio, or during talks with friends and family. Answers had to be indicated on 5-point Likert scales, ranging from 1 (“Very Rarely”) to 5 (“Very Often”; *α* = 0.80, *95%CI* [0.77, 0.83]).

#### Conspiracy theories beliefs

To measure participant’s beliefs about COVID19 conspiracy theories, participants were asked to indicate their level of agreement/disagreement with seven statements on a 5-point Likert scale, ranging their answers from 1 (“Disagree”) to 5 (“Agree”). As for previous research (see Pummerer et al., 2022), participants rated items such as “The COVID-19 pandemic is used as a population control scheme,” or “The COVID-19 virus is used by powerful people to crash the economy” (*α* = 0.95, *95%CI* [0.94, 0.96]).

#### Procedure

This second experiment also used Qualtrics and was conducted in English. After signing the informed consent and filling in demographic information, participants were asked to indicate their level of agreement with the seven items concerning the COVID-19 conspiracy theories. Next, participants were first exposed to the 6 news (i.e., 4 true and 2 fake), and asked to rate their memory for having heard/seen them. Furthermore, participants were asked to indicate where they have heard/seen the news from (i.e., TV, newspapers, radio, websites, social media, and family and/or friends), and how they felt about it.^8^ Then, participants were told that some of the news may have been fake, and they were asked to select which news they thought was fake (i.e., post-warnings^9^; see Murphy et al., 2019). Finally, participants completed the WordSum task, the CRT, and the interest and knowledge about COVID-19 questionnaires. In line with Experiment 1, the sequence of all the measures and questionnaires used-as well as the order of both true and fake COVID-19 news-were randomized across participants. Finally, participants were rewarded, thanked and debriefed.

### Results and discussion

#### True and false memory rate

Regarding true events, overall participants correctly recognized having heard/seen 2.40 (*SD* = 1.45) out of the 4 news stories presented. 45% (*n* = 148) of participants reported they have heard/seen these news mostly on the internet (25.9% websites, and 19.2% social media, respectively), followed by 26.2% (*n* = 86) from TV, 9.8% (*n* = 32) from family and/or friends, 7.3% (*n* = 24) from newspapers, 3% (*n* = 10) from the radio. The remaining participants (8.5%, *n* = 28) did not recall the source.

Furthermore, participants falsely recognized having heard/seen 0.82 (*SD* = 0.83) out of the 2 fake news stories presented, irrespective of having a conspiratorial content or not. Overall, 46.3% (*n* = 125) of participants claimed to having heard/seen the fabricated news from the internet (23.3% social media, and 23% websites, respectively), followed by 17.8% (*n* = 48) from TV, 10.4% (*n* = 28) from family and/or friends, 6.7% (*n* = 18) from newspapers, 4.8% (*n* = 13) from the radio. The rest of participants (14.1%, *n* = 38) did not recall the source.

Of interest, 54.9% (*n* = 185/337) of the sample indicated to remember at least one of the two fake news. More precisely, 52.1% (*n* = 88/169) and 34.5% (*n* = 58/168) falsely recalled the first (i.e., vaccines) and the second (i.e., controlling app) fake news with conspiratorial content, whereas 31% (*n* = 52/168) and 47.3% (*n* = 80/169) falsely recalled the first and the second fake news without conspiratorial content, respectively. Table 3 shows participants’ response for both true and fake news.

**Table 3:** Participants’ responses to true and fake news (Experiment 2).

Response	True news			
	Taiwan (*n* = 169)	Tiger Nadia (*n* = 168)	New York (*n* = 169)	Russia (*n* = 169)
I have a clear memory for seeing/hearing about this	27.2% (*n* = 46)	33.9% (*n* = 57)	55.6% (*n* = 94)	56.8% (*n* = 96)
I have a vague memory for this event occurring	7.7% (*n* = 13)	9.5% (*n* = 16)	4.1% (*n* = 7)	5.9% (*n* = 10)
I do not have a memory for this, but it feels familiar	14.8% (*n* = 25)	11.9% (*n* = 20)	11.2% (*n* = 19)	11.2% (*n* = 19)
I remember this differently	11.8% (*n* = 20)	11.3% (*n* = 19)	10.7% (*n* = 18)	8.9% (*n* = 15)
I do not remember this	38.5% (*n* = 65)	33.3% (*n* = 56)	18.3% (*n* = 31)	17.2% (*n* = 29)
Average true memory rating [mean (SD)]	0.34 (0.47)	0.43 (0.49)	0.59 (0.49)	0.62 (0.48)
	Bergamo (*n* = 168)	Trumps (*n* = 168)	Italy (*n* = 169)	Olympics (*n* = 168)
I have a clear memory for seeing/hearing about this	41.1% (*n* = 69)	77.4% (*n* = 130)	62.1% (*n* = 105)	64.9% (*n* = 109)
I have a vague memory for this event occurring	13.7% (*n* = 23)	5.4% (*n* = 9)	6.5% (*n* = 11)	8.9% (*n* = 15)
I do not have a memory for this, but it feels familiar	16.7% (*n* = 28)	3.6% (*n* = 6)	7.7% (*n* = 13)	6% (*n* = 10)
I remember this differently	7.1% (*n* = 13)	10.7% (*n* = 18)	12.4% (*n* = 21)	10.1% (*n* = 17)
I do not remember this	20.8% (*n* = 35)	3% (*n* = 5)	11.2% (*n* = 19)	10.1% (*n* = 17)
Average true memory rating [mean (SD)]	0.54 (0.49)	0.82 (0.37)	0.68 (0.46)	0.73 (0.44)
	Fake news			
	Conspiratorial content		No conspiratorial content	
	Vaccines (*n* = 169)	App (*n* = 168)	Vaccines (*n* = 168)	App (*n* = 169)
I have a clear memory for seeing/hearing about this	44.4% (*n* = 75)	25.6% (*n* = 43)	24.4% (*n* = 41)	40.2% (*n* = 68)
I have a vague memory for this event occurring	7.7% (*n* = 13)	8.9% (*n* = 15)	6.5% (*n* = 11)	7.1% (*n* = 12)
I do not have a memory for this, but it feels familiar	8.3% (*n* = 14)	13.7% (*n* = 23)	10.7% (*n* = 18)	12.4% (*n* = 21)
I remember this differently	16% (*n* = 27)	10.1% (*n* = 17)	25.6% (*n* = 43)	16.6% (*n* = 28)
I do not remember this	23.7% (*n* = 40)	41.7% (*n* = 70)	32.7% (*n* = 55)	23.7% (*n* = 40)
Average false memory rating [mean (SD)]	0.52 (0.50)	0.34 (0.47)	0.30 (0.46)	0.47 (0.50)

#### Conspiratorial beliefs and false memory

Based on participants’ responses to the conspiracy theory beliefs questionnaire, we divided them in two groups, namely people with conspiratorial beliefs (*n* = 165) or not (*n* = 172).^10^ On average, people who believed in conspiratorial theories to some extent agreed with 4.18 theories (*SD* = 2.09) out of the seven we showed them. More specifically, 84.24% (*n* = 139) of these people indicated to believe in more than one conspiratorial idea, 58.1% (*n* = 96) in more than three, and 34% (*n* = 56) in more than six. Of interest, these people agreed mostly with ideas such as “COVID-19 has been purposefully created” (64.85%, *n* = 107), “COVID-19 is used by powerful people to crash the economy” (64.25%, *n* = 106), and “COVID-19 is used as a biological weapon” (63.64%, *n* = 105).

We performed two Chi-square tests to examine the effect of conspiracy beliefs on false memory occurrence for both conspiratorial and non-conspiratorial COVID-19 fake news. People with conspiratorial beliefs were significantly more likely to remember COVID-19 fake news with conspiratorial content (65.6%) than people with no conspiratorial beliefs (31.5%), *χ^2^* (1, *N* = 300) = 34.74, *p* < 0.001, *Cramer’s V* = 0.34. Interestingly, the same group of people was even more likely to develop a false memory for COVID-19 fake news without conspiratorial content (61.6%) than the group with no conspiratorial beliefs (28.0%), *χ^2^* (1, *N* = 298) = 30.24, *p* < 0.001, *Cramer’s V* = 0.32. We replicated the same pattern of findings even when performing Chi-square tests for each COVID-19 fake news separately (all *p_s_* < 0.005). Finally, we did not reveal any statistically significant difference between these two groups with respect to true memory levels, *χ^2^* (4, *N* = 337) = 4.53, *p* = 0.338, *Cramer’s V* = 0.11., meaning that we deemed not relevant such a result.

#### Predictors of true and false memory for COVID-19 news

In line with previous work (Greene and Murphy, 2020), we ran linear regression and Poisson regression for both true and false memory rates, respectively, with predictor variables being (1) conspiracy beliefs (range: 0–7; *M* = 2.04; *SD* = 2.55), (2) cognitive abilities (range: 0–10; *M* = 5.33, *SD* = 2.73), (3) analytical thinking (range: 0–6; *M* = 2.53, *SD* = 2.04), (4) interest in COVID-19 (range: 7–35; *M* = 22.36, *SD* = 6.05), and (5) knowledge about COVID-19 (range: 1–10; *M* = 5.05, *SD* = 2.06). As for Experiment 1, we found both models to predict true, *R^2^* = 0.038, *F*(5,331) = 2.63, *p* = 0.024, and false memory, *χ^2^* (5) = 28.04, *N* = 337, *p* < 0.001, respectively. More precisely, we found cognitive abilities being marginally significant (*adjusted p* = 0.025) and, along with COVID-19 interest, related to an increased tendency to report true memories. Both factors yielded positive effect sizes. This means that for every 1-unit increase in cognitive abilities and COVID-19 interest levels, true memories increased by 1.075 and 1.035 units, respectively (see Table 4). The other factors (i.e., conspiracy beliefs, analytical thinking, and knowledge about COVID-19), were deemed not significant and bore negative effect sizes (see Table 4), meaning that we did not find relevant such associations. This suggests that for every 1-unit increase in those factors, true memories decreased by their corresponding value. Moreover, we showed that only lower analytical thinking levels were related to an increased tendency to report false memories, yielding a negative effect size [*exp(B)* = 0.859]. Note that the other factors of the model were deemed not significant, thus non-relevant, despite the positivity of their effect sizes (see Table 4).

**Table 4:** Regression coefficients for predictors of vulnerability to COVID-19 news (Experiment 2).

Predictor	*B*	*SE(B)*	*β^+^*	*t*	*p*	95% CI (*B*)	
						**Lower**	**Upper**
**True Memory Rate**							
(Constant)	1.23	0.405		3.05	0.002	0.442	2.03
Cognitive abilities	0.072	0.032	1.075	2.58	0.025	0.000	0.135
Analytical thinking	−0.031	0.041	0.969	−0.60	0.452	−0.112	0.050
COVID-19 knowledge	−0.012	0.044	0.987	0.196	0.773	−0.099	0.073
COVID-19 interest*	0.034	0.012	1.035	2.31	0.005	0.010	0.059
Conspiracy beliefs	−0.091	0.165	0.912	1.01	0.579	−0.415	0.232
	*B*	*SE(B)*	*β^+^*	*Wald χ^2^*	*p*	95% CI (*B*)	
						Lower	Upper
**False Memory Rate**							
(Intercept)	−0.683	0.079	0.505	0.710	0.001	−0.845	−0.531
Cognitive abilities	0.001	0.035	1.001	0.026	0.977	−0.070	0.070
Analytical thinking*	−0.151	0.052	0.859	8.66	0.004	−0.255	−0.048
COVID-19 knowledge	0.002	0.053	1.002	0.083	0.965	−0.102	0.105
COVID-19 interest	0.007	0.015	1.007	0.321	0.636	−0.022	0.036
Conspiracy beliefs	0.274	0.207	1.316	0.131	0.187	−0.133	0.679

When performing several tests, we applied the Bonferroni adjustment to reduce the likelihood of generating false-positive results (type I errors).*Bonferroni adjustment *p* < 0.01; ^+^For linear regressions (i.e., true memory rate), β is given as 0 for no effect with values <0 for negative effects and >0 for positive effects. Whereas for Poisson regressions (i.e., false memory rate), β [i.e., exp(B)] is given as 1 for no effect, with values >1 for positive effects and <1 for negative effects.

To recap, as for Experiment 1, more than half of our sample (≈ 55%) reported at least one false memory for a COVID-19 fake event that has never took place. Irrespective of its content, people with conspiratorial beliefs reported more false memories for COVID-19-related fake news than those without conspiratorial beliefs. Even though research has indicated that people are more vulnerable to misinformation when this matches their pre-existing attitudes or beliefs (Frenda et al., 2013; Greenspan and Loftus, 2021), in our experiment conspiratorial beliefs concerning COVID-19 seemed to amplify people’s susceptibly to false information in general, making them more prone to believe both conspiratorial and non-conspiratorial materials.

With respect to our regression models, only lower analytical thinking was associated with COVID-19 false memory formation. Specifically, but in contrast with some previous work showing no correlation between CRT and false memory (see Nichols and Loftus, 2019; Scuotto et al., 2021) participants with lower CRT scores were more likely to report a COVID-19 fake event. This result further highlights the idea that good analytical thinking skills are important to spot fake news (Greene and Murphy, 2020). By contrast, cognitive abilities were positively linked to true memories, but were not associated with false memory rates. Along with this, also high level of COVID-19 self-interest were associated with reporting more true news, suggesting therefore that being exposed to or engaged in discussion on COVID-19 could enhance correct memory on such a topic.

## General discussion

In two experiments, we examined whether people were likely to form false memories for COVID-19-related fake news. Furthermore, we investigated which factors underpinned the relation between fake news and false memory formation. We now discuss findings from Experiments 1 and 2 first with respect to false memories for COVID-19-related news, and then concerning individuals’ variables underling such a false memory formation.

Overall, many people fell prey to COVID-19-related fake news (41% in Experiment 1; 54.9% in Experiment 2). The digital climate we live in likely amplifies the vulnerability to false memory. Exposure to and profusion of COVID-19 fake news, for instance through social media, not only has consequences on people’s behaviours (Ahmed et al., 2020) but also on their memory (Greenspan and Loftus, 2021). Participants were fairly poor at recognizing fabricated events, and this effect was even more evident in Experiment 2. Such an effect, however, might have to do with when COVID-19 materials referred to widespread fake news. That is, in Experiment 2, we exposed participants to fake news concerning negative effects of vaccination and COVID-19 tracking apps. These two examples underwent several heated discussions on social media and among members of the general population. Arguably, the context of these fake news was quite known to our participants and yet they failed to discriminate them as such.

Relatedly, in Experiment 2, in contrast with those who did not believe in conspiracy theories, people with conspiracy beliefs were more likely to report a false memory for a fabricated event irrespective of its conspiratorial content. As recently shown (e.g., Anthony and Moulding, 2019), people with conspiracy beliefs are commonly more inclined to believe in fabricated events. So, in line with prior work indicating that conspiracy theories significantly correlated with mistrust in scientists and governments, the nature of the two fake news (i.e., effects of the vaccines and tracking apps) might have led participants who believe in conspiracy theories to over-generally rate them as true, thereby making source monitoring misattributions.

Our two experiments examined several variables that could predict false memory formation. In Experiment 1, well-being was positively associated with a tendency to report memories for COVID-19 events, whether they were true or false. To some extent this may indicate a response bias which may have inflated participants’ overall recognition, hence enhancing acceptance rates for both true and false news stories. Alternatively, moderate well-being levels might be linked to a general vulnerability to accepting COVID-19 materials. It may be the case that virus-related anxiety may represent a more nuanced predictor of individuals’ resistance to reporting false memories as compared with how people generally feel during the pandemic, even though some other factors may come into play (e.g., people’s ambiguity aversion or personal need for structure). This means that a general emotional distress generated by the pandemic might affect people’s way to discern COVID-19 fabricated news from true news, in the sense that such a distress made participants more attentive to information about COVID-19, somehow “activating” them towards specific news related to their distress. Still, the relationship between overall people’s well-being, as well as more specific health-related affective states (e.g., anxiety, depression; Scuotto et al., 2021), and false memory formation related to COVID-19 fake news should be further investigated in future studies.

Moreover, in Experiment 2, lower analytic thinking predicted false memory formation. In line with Greene and Murphy (2020; but see Scuotto et al., 2021), this result suggests that while determining whether a true memory for an event is present, higher analytic thinking skills might protect individuals from reporting a false memory for a fabricated event. Thus, this finding further underlines the importance of critical thinking abilities in resisting fake news and misinformation (Pennycook and Rand, 2019; Greene and Murphy, 2020). However, we did not find support for the fact that lower cognitive ability would result in an increased susceptibility to false memories in contrast with previous research (e.g., Zhu et al., 2010; Murphy et al., 2019). Note that, however, only one study indicated that lower levels of cognitive ability [measured with the Wordsum Test (Wechsler, 2008)] predict false memory for COVID-19 fabricated events, but only when associated with an increased effect of ideological congruence on false memories (Murphy et al., 2019). This might indicate then that when considering cognitive ability alone, this factor does not have a direct effect on false memory production, but it may work as a moderator on the effect of ideological congruence on false memories formation. Equally, self-interest (i.e., engagement with the topic of COVID-19) was not identified as was not identified as significantly predicting false memory formation. This factor was instead associated with a tendency to report memory for true news, confirming that showing interest towards COVID-19 could improve proper recollection about related issues.

Several limitations of the current research should be mentioned. We cannot rule out the possibility that exposing participants to COVID-19 negative news might have conflicted with their task performance (i.e., recognition). A recent study showed that people process negative and neutral COVID-19 materials differently, and this influenced their performance on the tasks, along with their affective states (see Ribatti et al., 2022). Because we did not differentiate COVID-19 news based on their valence, we cannot fully disentangle the role that this latter played on false memory formation. Additionally, our fake news stories could have strengthened pre-existing false memories, if participants were already exposed to similar, if not identical fake news during the ongoing pandemic. Take for instance the fake news adopted in Experiment 1. During the pandemic, several images with piled-up coffins circulated in the media. While some of these came from reliable sources, some others were eventually recognized as fake (e.g., the infamous Italian photo taken from the Lampedusa’s accident used to mistakenly display deaths due to COVID-19). Similarly, several media reported that some people found restrictive measures excessive. This information overlapped with a portion of our second fake news. Hence, we cannot fully determine whether our fabricated news made people reporting false memory for the events *ex novo*, or simply reinforced an existing one (Greene and Murphy, 2020). Hence, we cannot fully determine whether our fabricated news made people reporting false memory for the events *ex novo*, or simply reinforced an existing one (Greene and Murphy, 2020). Finally, because we developed both COVID-19’s knowledge and self-interest measurements, perhaps these tools did not entirely access participants’ dimensions concerning COVID-19’s interest and knowledge, respectively. This, in turn, could have affected our results. Hence, it would be important in future studies to dig into people’s COVID-19’s interest and knowledge applying measures that could better reflect these constructs and their association with false memory formation. In order to do so, for instance, it would be worth it to endorse a “knowledge calibration” tool, thereby finding a common, and better ground between one’s self-assessed and existent knowledge (Scuotto et al., 2021).

In closing, our findings demonstrated that people can easily report false memory for COVID-19-related fake news, along with true memories. In both experiments, participants did not discriminate between true versus false contents when recalling COVID-19-related news. Furthermore, people who believed in conspiracy theories were more likely to remember fabricated news, irrespective of their content. Also, false memory formation was likely among people who showed less well-being and analytical reasoning. In addition to similar work (Frenda et al., 2013; Murphy et al., 2019; Greene and Murphy, 2020; Greene et al., 2021), it is imperative to stress that our findings have several implications. Considering the role that digital contexts (e.g., social media platforms) play in our lives, exposure to fake news is omnipresent (see Marco-Franco et al., 2021). Although research into fake news is relatively in its infancy (Nyilasy, 2019), the COVID-19 pandemic has revealed how powerful misleading information about this event can spread through society. Nowadays, people spend much time on social media, reading and sometimes spreading health-related fake news that can affect people’s memories, and consequently their behaviours (see Ahmed et al., 2020). For instance, several fake news stories circulated around the effects of vaccines. Some of these were recycled from old conspiracy arguments (e.g., vaccines contain dangerous metals such as aluminium salts), while others were relatively novel (e.g., vaccines contain controlling microchip). Either way, reading, remembering, sharing, and acting upon those fake news has – among other reasons – detrimentally affected the vaccinations’ campaigns in some countries (Chen et al., 2022). Hence, targeted interventions could be implemented both by governments and social media companies (e.g., health and critical thinking training in school curricula; Frechette, 2019; and fact-checking, warnings; Greenspan and Loftus, 2021, respectively), to debunk COVID-19 misinformation, and help people to independently and correctly discern true and fake news.

## Data availability statement

The original contributions presented in the study are included in the article/Supplementary material, further inquiries can be directed to the corresponding author.

## Ethics statement

The studies involving human participants were reviewed and approved by Sociaal-Maatschappelijke Ethische Commissie (SMEC), KU Leuven. The patients/participants provided their written informed consent to participate in this study.

## Author contributions

HO, FB, EC, and NK conceived and designed the experiments. EC and NK collected the data. IM analysed the data, with FB’s assistance. IM wrote the first draft of the manuscript. FB, TW, AC, and HO added to and edited the manuscript. All authors contributed to the article and approved the submitted version.

## Funding

The current article has been supported by a C1 grant (Application number: C14/19/013) from the KU Leuven (Belgium) and a FWO Senior Research Project grant (Application number: G0D3621N; Research Foundation – Flanders, Belgium) awarded to the last author. This research has also been conducted under the National Operational Plan (PON) project “Ricerca e Innovazione” 2014-2020 (Number: 737-H95F21001470001; Minister of University and Research, Italy) granted to IM.

## Conflict of interest

The authors declare that the research was conducted in the absence of any commercial or financial relationships that could be construed as a potential conflict of interest.

## Publisher’s note

All claims expressed in this article are solely those of the authors and do not necessarily represent those of their affiliated organizations, or those of the publisher, the editors and the reviewers. Any product that may be evaluated in this article, or claim that may be made by its manufacturer, is not guaranteed or endorsed by the publisher.

## Footnotes

^1^5G is an information technology that allows for faster mobile internet communication and will allow for new innovative services.

^2^Twitter is a social networking service on which people post and interact with instant messages known as "tweets."

^3^Qualtrics is a business operating system that captures and stores data from customers in a single system of record (www.qualtrics.com).

^4^We did not report results about COVID-19 point of view because of the discrepancy between the number of participants who consider COVID-19 as a serious matter and stick to the governments’ restrictive measures and those who did not (*n* = 143 vs. *n* = 11, respectively). However, results can be found on OSF (osf.io/3xhkt).

^5^We chose linear and Poisson regression to model the count variable of true and false memories, respectively, because they offered a better fit to the data. The assumptions of the analysis were met by looking at "the goodness of fit" using the sums of squares, residuals vs. predicted, and Q-Q plot standardized residuals.

^6^MTurk is a sourcing model website often adopted to hire people to perform tasks remotely. Participants recruited *via* MTurk for the current survey had a HIT approval rate (i.e., proportion of completed tasks that are approved by requesters) of 98% and more than 5,000 HITs approved.

^7^What we meant to convey with our fake news was that serious side-effects were the most likely outcome during the vaccination trials.

^8^On OSF (osf.io/3xhkt), we reported frequencies analysis about participants’ feelings at the moment they have heard/seen both true and fake news.

^9^Because beyond the scope of the paper, we reported analyses concerning post-warnings on OSF (osf.io/3xhkt).

^10^Participants who showed agreement (i.e., "Somewhat agree" or "Agree") to at least one of the item regarding the conspiracy theory beliefs questionnaire were deemed as people believing in conspiracy theory.
