# Supplementary material for: False memory and COVID-19: How people fall for fake news about COVID-19 in digital contexts
Source: Front Psychol. 2022 Oct 13;13:972004. doi: 10.3389/fpsyg.2022.972004 (PMC9608342; doi:10.3389/fpsyg.2022.972004)
Supplement: Supplementary file 1 [file Table_1.DOCX]

**Supplemental Materials**

**SM1: True news adopted in Experiment 1**

1. Belgian Virologist Marc van Ranst has lashed out via Twitter^[[1]](#footnote-1)^ at people who underestimate or deny the seriousness of COVID-19. According to him, the second wave was partly a result of such a thinking: “Here in Belgium, too, we lost precious time in the early stages of the second wave by ‘second-wave deniers’ who portrayed the virologists as panic mongers. The virologists were right. The second wave deniers were wrong. Ok, let's move on”.
2. An 8-year-old Belgium child underwent intensive care because affected by COVID-19. To make other people aware about the gravity of the pandemic, the father of the 8-year-old child posted his son’s picture on Facebook^[[2]](#footnote-2)^: “Anyone who thinks that COVID-19 is nonsense can come and have a look at my poor son”.
3. Rush Limbaugh, who received the Presidential Medal of Freedom from Donald Trump, said on his radio show on February the 24^th^ that ‘The virus is no worse than a cold. It seems like COVID-19 is being used as a weapon and an additional argument to bring down Donald Trump”; “But the coronavirus is just a common cold, folks”.
4. Brazil’s President, Jair Bolsonaro, called the fear of COVID-19 “hysterical” and wanted administrators to reverse the measures in his country. He said “We have to go back to the normal situation”. He had previously seen the virus as “fantasy”, comparable to a “minor flu”. He also added he would not feel anything if he caught the virus.

**SM2: True news adopted in Experiment 2**

1. Thanks to a new technological development, Taiwan has achieved to reduce the numbers of COVID-19’s infections. A “mobile phone-based electronic fence app”, applying location tracking, helps to make sure that quarantined people do not leave their houses. In case a quarantined citizen violates the quarantine-regulations and leaves the home area, the system sends alerts to police and local officials.
2. In April, the coronavirus had been discovered in a wild animal. Testing Tiger Nadia, living in Bronx zoo (New York), for the virus that causes COVID-19, has yielded a positive result. It is presumed, that this tiger might have been infected by one of the zookeepers.
3. New York City’s hospitals have been overwhelmed with coronavirus cases. This is why a US navy hospital ship docked in New York city harbour. This had not happened since 9/11.
4. As a first country, Russia, has announced the registration of a COVID-19 vaccine in August. Many scientists reacted with scepticism, since test phase three, usually lasting over months and requiring thousands of test persons, had not yet been completed.
5. Crematoriums in Bergamo (Italy) had been overwhelmed with the number deaths from COVID-19. Consequently, military trucks helped to transport numbers of coffins out of Bergamo to other crematoriums in surrounding cities.
6. Donald Trump and first Lady, Melania Trump, tested positive for COVID-19 approximately 1 month prior to the Election Day.
7. Many people in Italy spontaneously started making music for, and together with, neighbours on their balconies during the lock-down to combat the “virus-blues” and the isolation.
8. As a result of the COVID-19 pandemic, the Olympic Games had to be postponed. This had never happened before in history.

1. Twitter is a social networking service on which people post and interact with instant messages known as "tweets". [↑](#footnote-ref-1)
2. Facebook is a social networking site that makes it easy for people to connect and share with others online. [↑](#footnote-ref-2)
